# Molecular characterization of *Blastocystis* subtypes isolated in the city of Uberaba, Minas Gerais State, Brazil

**DOI:** 10.1590/0037-8682-0305-2021

**Published:** 2021-08-20

**Authors:** Marlene Cabrine-Santos, Renata Gregório Franco Moura, André Luiz Pedrosa, Dalmo Correia, Márcia Benedita de Oliveira-Silva

**Affiliations:** 1 Universidade Federal do Triângulo Mineiro, Instituto de Ciências da Saúde, Departamento de Biomedicina, Uberaba, MG, Brasil.; 2 Universidade Federal do Triângulo Mineiro, Programa de Pós-graduação em Medicina Tropical e Infectologia, Uberaba, MG, Brasil.; 3 Universidade Federal do Triângulo Mineiro, Instituto de Ciências Biológicas e Naturais, Departamento de Bioquímica, Farmacologia e Fisiologia, Uberaba, MG, Brasil.; 4 Universidade Federal do Triângulo Mineiro, Instituto de Ciências da Saúde, Departamento de Clínica Médica, Uberaba, MG, Brasil.; 5 Universidade Federal do Triângulo Mineiro, Instituto de Ciências Biológicas e Naturais, Departamento de Microbiologia, Parasitologia e Imunologia, Uberaba, MG, Brasil.

**Keywords:** *Blastocystis* species, Subtype, Genetic characterization, Brazil

## Abstract

**INTRODUCTION:**

*Blastocystis* is an intestinal protozoan that may play a role in the pathogenicity of humans. This study aimed to (i) genetically characterize *Blastocystis* isolates obtained from human fecal samples and the water supply of the city of Uberaba, Minas Gerais, Brazil, and (ii) to verify the phylogenetic relationship between these isolates.

**METHODS:**

*Blastocystis* species present in 26 fecal samples obtained from humans and animals from Uberaba were genetically characterized by polymerase chain reaction-restriction fragment length polymorphism and polymerase chain reaction-sequence-tagged sites. All amplicons were partially sequenced and/or defined according to the GenBank classification.

**RESULTS:**

Polymerase chain reaction amplicons were generated from 21 human isolates and 18 water samples. The subtypes defined were ST1 (53.3%), ST3 (40.0%), and ST2 (6.7%) for human isolates; ST10 (100%) for bovine isolates; and ST5 (50.0%), ST1 (25%), and ST3 (25%) for pigs. Sequencing of polymerase chain reaction products showed a 98%-99% identity for the *Blastocystis* sequences deposited in GenBank, except for sequences from water samples that showed the identity of algae sequences. Phylogenetic analysis of *Blastocystis* sequences showed two distinct groups, one of which was principally formed by ST1, ST5, and ST10, and the other by isolates characterized as ST3 and ST7. Both clades showed human and animal sequences, reinforcing the notion that *Blastocystis* subtypes are not host-specific.

**CONCLUSIONS:**

The data showed that *Blastocystis* subtypes circulating in Uberaba are ST1-ST3, ST5, and ST10, present in both humans and animals, demonstrating that the *Blastocystis* subtypes are not host-specific; that is, zoonotic transmission is possible.

## INTRODUCTION

*Blastocystis* is a Stramenopile of the Blastocystidae family with a cosmopolitan distribution; they are the most prevalent parasites found in human feces worldwide despite the distinction between their colonization and infection not being well distinguished thus far^1^ ^-^ ^4^. Literature suggests that the parasite can interact with the host's microbial flora; however, the consequences of this interaction are not yet well known^4^. 

Although underestimated, the prevalence of *Blastocystis* spp. in human hosts ranges from 17.8% to 86.63% in Brazil, as estimated by the diagnostic methods employed and the technical ability of laboratory technicians for its recognition^1^ ^,^ ^5^ ^,^ ^6^. 

The molecular characterization of *Blastocystis* isolates has been carried out using different techniques; however, the analysis of the small ribosomal subunit gene (SSU-rDNA) is the most commonly used method^7^ ^-^ ^11^. These studies showed that *Blastocystis* has broad genetic diversity and is classified into 17 subtypes (ST1-ST17^12^). Ten *Blastocystis* subtypes have been described in humans: ST1-ST9 and ST12^4^ ^,^ ^10^ ^,^ ^12^. Other subtypes have been found in pets, livestock, and zoo animals^12^. 

Several studies have attempted to establish a relationship between *Blastocystis* subtype and clinical symptoms in patients; however, this relationship remains inconclusive^13^ ^,^ ^14^. Humans are mainly infected by ST1-ST4 and rarely by ST5-ST9^4^ ^,^ ^12^ ^,^ ^15^ ^-^ ^19^ ^,^ ^20^.

In distinct regions of Brazil, different authors have demonstrated the presence of ST1-ST4 and ST6-ST8 subtypes. The ST1-ST3 and the ST2 subtype are the most prevalent^21^ ^-^ ^25^. In the city of Uberaba, Minas Gerais (MG), an area of the Brazilian savannah, the parasite has been observed in humans (~17%), pigs (72.2%), sheep (33.3%), cattle (21.4%), and dogs (2,3%)^6^ ^,^ ^26^. However, the molecular epidemiological profile of *Blastocystis* isolated in this region remains unknown.

This study aimed to: (i) genetically characterize the isolates of *Blastocystis* species obtained from human fecal samples and water supply in the city of Uberaba, MG, Brazil, and (ii) verify the phylogenetic relationship between *Blastocystis* isolates from humans, pigs, cattle, and water, to define the epidemiological profile of the parasite in this region.

## METHODS

### *Blastocystis* samples

In this study, *Blastocystis* species present in 26 fecal samples obtained from Uberaba, MG, Brazil, were genetically characterized. Of these, 21 samples were of human origin^6^, four pig feces, and one bovine^26^. In addition, the presence of *Blastocystis* DNA was tested in 18 water supply samples^27^ in the same area.

### DNA extraction and PCR

Total DNA was extracted from the fecal samples using the immunomagnetic Magnex DNA Kit (Labtest Diagnóstica S.A., Minas Gerais, Brazil) according to the manufacturer’s instructions and protocol described by Moreira et al.^27^. Samples were screened for *Blastocystis* by polymerase chain reaction-restriction fragment length polymorphism (PCR-RFLP), as previously described^28^. Briefly, the SSU rDNA gene fragment amplified by PCR with primers 1FB and 1RB (Table 1) was *Spe*I-digested to infer parasite classification^28^. PCR-sequence-tagged site (PCR-STS) analysis was performed, and the subtypes were determined^7^ ^,^ ^29^.

Bovine and pig samples were obtained from another study by our team^26^. The authors classified the S03 pig sample as ST1 using the same methods used in the present study. However, the authors could not determine the subtypes of the pig samples S01, S02, and S06, or bovine sample B01; nonetheless, they were sequenced.

**TABLE 1:** Primers used to amplify the Small Subunit rDNA gene of *Blastocystis* isolates of humans, animals, and the water supply from the state of MG Brazil.

Primer	Sequence (5′→3′)	Size (bp)	Reference
1FB	GGAGGTAGTGACAATAAATC	1100	Yoshikawa et al. (2000)
1RB	ACTAGGAATTCCTCGTTCATG		
SSU907 F-BH	TGAAACTGCGAATGGCTCA	907	This study
SSU907 R-BH	CAAGAACGAAAGCTAGGGGA		
SSU850 F-BH	GCGAAAGCATTTACCAAGGA	850	This study
SSU850 R-BH	CCTACGGAAACCTTGTTACGA		

### DNA sequencing and analysis

All amplicons from human and supply water samples were partially sequenced. To obtain the sequence of the entire SSU rDNA gene fragment, two pairs of internal primers (Table 1) were designed, and two additional PCRs were performed, namely PCR-INT 1 and PCR-INT 2, amplifying 907 bp and 850 bp fragments of the SSU rDNA gene, respectively; the amplified products were sequenced. All sequencing reactions were performed using the ABI Prism BigDye Terminator version 3.1, Cycle Sequencing Kits® (Applied Biosystems, Inc., Grand Island, USA) and analyzed using the ABI Prism 3500 Genetic Analyzer (Applied Biosystems). The nucleotide sequences were curated, and the consensus sequence of each sample was generated using the ChromasPro® version 1.7.6. 

SSU-rDNA PCR fragments of some *Blastocystis* isolates from humans (n=7), pigs (n=3), and bovines (n=1)^26^ were not classified by PCR-RFLP and PCR-STS; however, they were sequenced. To mitigate this problem, these sequences were aligned using data regarding *Blastocystis* present in the GenBank database. With sequence alignment greater than 95% and an error less than 5%, the subtypes were defined according to the Genbank classification based on the Stensvold and Clark (2016) statement^4^. 

The similarity analysis of the sequence generated with GenBank data was verified, and phylogenetic analysis was performed using the Mega® version 6 software^30^, based on the SSU rRNA dataset of *Blastocystis* by the 95 Maximum Likelihood method (ML) with 1000 bootstrap replicates. Reference sequences obtained from *Blastocystis* isolates ST1 (MK801358), ST3 (MK801403), ST5 (MK801414), ST7 (AF408427), and ST10 (MH507326) were used to make comparisons in dendrogram analysis; thus, an outgroup represented for *Thecamonas trahens* (XR_001290156)*.*

### Ethical approval

The project was approved by the Universidade Federal do Triângulo Mineiro’s Research Ethics Committee, Avenida Getúlio Guaritá, 159, Casa da Comissões, Bairro Abadia, Uberaba/MG, CEP: 38.025-440, phone +55 (34) 3700-6803, e-mail: cep@uftm.edu.br, under protocol number 1804. 

## RESULTS

PCR amplicons of 21 isolates from human fecal samples and 18 isolates from water samples were generated; however, these had different sizes (0.9 to 1.3 kb) than expected (1.1 kb) in 18/21 isolates from humans and 17/18 water isolates. 

PCR-RFLP showed the restriction profiles of 14 human isolates (Table 2). After performing the PCR-STS following the generated profile, eight subtypes of *Blastocystis* were defined: five as ST1, two as ST3, and one as ST2 (Table 2). Three samples did not have sufficient DNA for PCR-STS analysis, and three were not amplified.

**TABLE 2:** *Blastocystis* subtypes determined by molecular methods from human feces samples in the city of Uberaba, MG State, Brazil.

***Blastocystis* isolates**	PCR-RFLP of SSU-rDNA*		SSU-rDNA GenBank accession number	Subtype inferred by sequence comparison^†^	Identity (%)	PCR-STS
	SSU-rDNA amplification	SpeI digestion				
H09	Yes	ND	KX257271	ST1	99.1-99.9	-
H31	Yes	ST1, ST2	KX257272	ST1	99.1-99.9	ST1
H38	Yes	ST1, ST2	KX257273	ST1	99.1-100.0	ST1
H40	Yes	ND	KX257274	ST3	97.0-98.9	-
H42	No	-	-	-	-	ST3
H46	Yes	ST1, ST2	KX257275	ST1	99.1-100.0	ST1
H177	Yes	ND	KX257283	ST3	97.0-99.2	-
H212	Yes	ND	KX257276	ST3	97.0-99.2	-
H216	Yes	ND	KX257277	ST3	97.0-99.6	-
H366	Yes	ND	KX257278	ST1	99.6-99.8	-
H495	Yes	ST3, ST4, ST8/ST5, ST7	KX257279	ST3	97.1-99.9	ST3
H496	Yes	ST1, ST2	KX257280	ST1	98.0-100.0	ST1
H543	Yes	ST1, ST2	KX257281	ST1	99.1-100.0	ST1
H595	Yes	ST1, ST2	-	-	-	ST2
H621	Yes	ND	KX257282	ST1	99.6-99.8	-
B01	Yes	ND	KX257266	ST10	97.0-98.5	-
S01	Yes	ND	KX257267	ST5	98.1-98.5	-
S02	Yes	ND	KX257268	ST5	98.5-98.7	-
S03	Yes	ST1^‡^	KX257269	ST1	98.4-100.0	ST1
S06	Yes	ND	KX257270	ST3	97.6-98.2	-
Ref.Bsp_ST1	-	-	MK801358	ST1	98.4-100.0	-
Ref.Bsp_ST3	-	-	MK801403	ST3	97.4-99.4	-
Ref.Bsp_ST5	-	-	MK801414	ST5	99.1-99.9	-
Ref.Bsp_ST7	-	-	AF408427	ST7	97.7-99.4	-
Ref.Bsp_ST10	-	-	MH507326	ST10	95.8-99.7	-

*PCR-RFLP: *Polymerase Chain Reaction- Restriction Fragment Length Polymorphism. SSU rDNA: Small Ribosomal Subunit*; SpeI Restriction endonuclease (Yoshikawa et al., 2011).

^†^According to Stensvold and Clark (2016), *Blastocystis* subtypes can be inferred when the comparison of their sequence with the GenBank sequences has an alignment > 95% and error < 5%.

‡Sample classified as ST1 in the study of Moura et al. (2018).

**ND:** Not determined.

Seven *Blastocystis* isolates from humans did not show a restriction profile after performing PCR-RFLP, probably because of the small amount of amplified DNA. In this case, PCR-STS was performed with all primer pairs to define each subtype of *Blastocystis* (ST1-ST7); however, no sample was amplified, and the parasite subtype could not be defined. 

Amplicons obtained from *Blastocystis* isolates from water samples did not show any restriction profile after PCR-RFLP, and the DNA was not amplified by PCR-STS.

In this study, high-quality sequences of 13 isolates from human fecal samples, 1 of an isolate from a bovine sample, 4 of isolates from pig samples (Table 2), and 4 of isolates from water samples were generated. However, analysis of the water sequences showed that the products amplified by PCR corresponded to the DNA of the algae, Eustigmatophyceae, and not to *Blastocystis*, thus showing that this PCR is not suitable for environmental samples.

**FIGURE 1:**
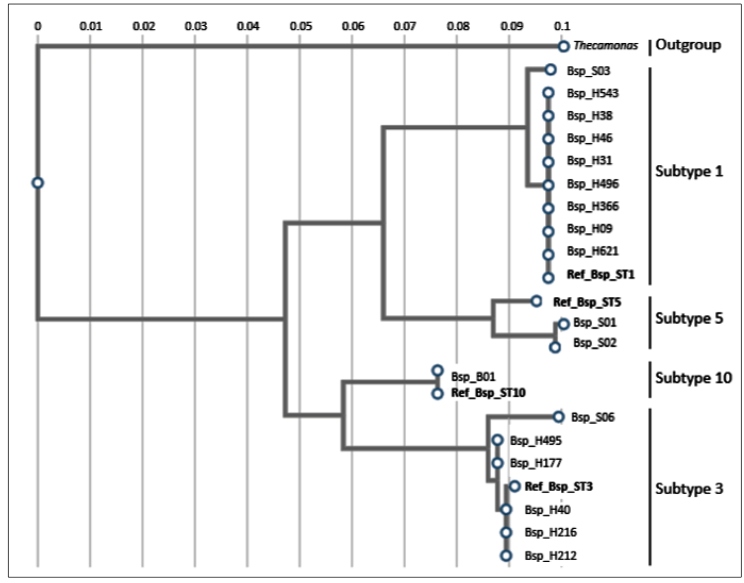
Dendrogram obtained by multiple alignments of DNA sequences generated from *Blastocystis* species from humans and animals in the Uberaba city, MG, Brazil. A segment of the 18S rDNA gene of *Thecamonas trahens* (ATCC 50062, accession number XR_001290156) was used as an outgroup. Phylogenetic analysis was realized by Mega® version 6 software30, based on the SSU rRNA dataset of *Blastocystis* by the 95 ML method with 1000 replicates bootstrap values. Ref: reference strain; Bsp: *Blastocystis* spp.

Sequences obtained from human fecal samples (KX257271 to KX257283) and animals (KX257266-KX257270) were deposited in GenBank. Comparison of these sequences with GenBank data showed polymorphism of the SSU rDNA gene, showing substitutions in some regions and insertions or deletions (indel events) in others ( >Figure S1). Additionally, this analysis allowed the inference of the subtypes of three isolates of *Blastocystis* from pigs (2 ST5, 1 ST3), one from bovine (ST10), and seven from humans (3 ST1, 4 ST3) not classified by PCR-STS as described in the Methods section (Table 2, Figure 1). 

In summary, in this study, the subtypes defined for human isolates were ST1 (8/15, 53.3%), ST3 (6/15, 40.0%), and ST2 (1/15, 6.7%); ST10 for the bovine isolate; ST5 (2/4, 50.0%), ST1 (1/4, 25%), and ST3 (1/4, 25%) for the pig isolates.

Phylogenetic analysis of *Blastocystis* sequences showed two distinct groups, one of which was principally formed by the isolates characterized as ST1, ST5 (S01, S02) and ST10 (B01). Another group was formed with isolates characterized as ST3 and ST7 (Figure 1). 

## DISCUSSION

In the present study, we confirmed that the SSU rDNA gene is polymorphic, as DNA products of different sizes from the described 1.1 kb fragment^29^ were amplified in human feces, indicating a similarity of approximately 99% with the *Blastocystis* sequences deposited in GenBank. Several indel events were observed in the generated sequences. 

These results showed ST1-ST3, ST5, and ST10 in the city of Uberaba, with ST5 and ST10 present only in pigs and cattle, respectively. ST1 and ST3 were isolated from both humans and pigs, demonstrating the zoonotic potential of transmission of *Blastocystis* species, as reported by other authors^31^ ^-^ ^34^.

Few studies have been carried out in Brazil regarding the geographic distribution of *Blastocystis* subtypes and their hosts^35^. ST1-ST4 have been reported to date, with a predominance of ST1 and ST3^21^ ^,^ ^26^ ^,^ ^35^ ^-^ ^36^. In the world panorama, the most prevalent subtypes of *Blastocystis* spp. are ST1-ST4, but ST5-ST9 and ST12 have also been described and have different regional prevalence^37^ ^-^ ^39^. Mixed infections have also been reported^11^, showing that complex genotypes may occur in various regions of the world.

Phylogenetic analysis showed that sequences originating from humans formed two distinct groups, one of which contained the sequences of the isolates characterized as ST1 and the other with the isolates characterized as ST3. In recent studies^22^ ^,^ ^35^, the grouping of subtypes into separate clades was shown by phylogenetic analysis of the different *Blastocystis* subtypes observed in Brazil in the South, Southeast, and Midwest regions. Pig isolates, although more similar to each other than human isolates, were divided into clades of the dendrogram, either isolated or belonging to one of the groups of human isolates. These data reinforce the potential for zoonotic transmission^22^ ^,^ ^35^ ^,^ ^40^. 

In our study, 4 of the 18 water samples amplified with *Blastocystis*-specific primers followed by sequencing did not correspond to sequences of this parasite. Other studies in Brazil using PCR with primers other than those we used showed that amplified DNA fragments of water samples from the Tietê River, State of São Paulo, also did not correspond to the *Blastocystis* sequences deposited in GenBank^35^. These results show that the primers directed to rDNA targets, which are highly conserved regions among eukaryotes, are inadequate for investigating *Blastocystis* in environmental samples. Consequently, the use of primers directed to these regions is a limitation for investigating *Blastocystis* in environmental samples; their use in PCR tests may contribute to false-positive results if other techniques, such as DNA sequencing, are not employed. These observations point to the need for developing new specific primers and/or new techniques to investigate the presence of *Blastocystis* in environmental samples. 

Based on our study, we concluded that the SSU rDNA gene is polymorphic and may define intraspecific variations leading to the grouping of the isolates according to their genetic characteristics. In addition, it was verified that in the studied region, the subtypes of *Blastocystis* were ST1, ST3, and ST2, and zoonotic transmission is possible, as ST1 was found in both humans and pigs in the region. Finally, it was observed that the PCR of the SSU rDNA gene was not useful for the detection of *Blastocystis* in environmental samples, as it amplified the non-specific DNA of algae.
