# Supplementary material for: Molecular characterization of Blastocystis subtypes isolated in the city of Uberaba, Minas Gerais State, Brazil
Source: Rev Soc Bras Med Trop. 2021 Aug 20;54:e0305-2021. doi: 10.1590/0037-8682-0305-2021 (PMC8405216; doi:10.1590/0037-8682-0305-2021)
Supplement: Supplementary file 1 [file 1678-9849-rsbmt-54-e0305-2021-supp1.pdf]

Supplementary Figure 1

```
KX257266_B01          AAGATTAAAAGGGACAGTTGGGGGTATTCATATTCAATAGTCAGAGGTGAAATTCTCGGA   60
MH507326_Bsp_ST10     AAGATTAAAAGGGACAGTTGGGGGTATTCATATTCAATAGTCAGAGGTGAAATTCTCGGA   60
KX257270_S06          AGATTAAAAGGAACAGTCGGGGCGTATTCATATTCACTAGTTAGAGGTGAAATTCTCGGA   60
KX257274_H40          AAGATTAAAAGGGACAGTTGGGGGTATTCATATTCAATAGTCAGAGGTGAAATTCTCGGA   60
KX257283_H177         AAGATTAAAAGGGACAGTTGGGGGTATTCATATTCAATAGTCAGAGGTGAAATTCTCGGA   60
KX257276_H212         AAGATTAAAAGGGACAGTTGGGGGTATTCATATTCAATAGTCAGAGGTGAAATTCTCGGA   60
KX257277_H216         AAGATTAAAAGGGACAGTTGGGGGTATTCATATTCAATAGTCAGAGGTGAAATTCTCGGA   60
KX257279_H495         AAGATTAAAAGGGACAGTTGGGGGTATTCATATTCAATAGTCAGAGGTGAAATTCTCGGA   60
MK801403_Bsp_ST3      AAGATTAAAAGGGACAGTTGGGGGTATTCATATTCAATAGTCAGAGGTGAAATTCTCGGA   60
AF408427_Bsp-ST7      AAGATTAATAGGGACAGTTGGGGGTATTCATATTCAATAGCTAGAGGTGAAATTCTATGA   60
KX257271_H09          AAGGTTAAAAGGAACAGTTGGGGGTATTCATATTCACTAGTTAGAGGTGAAATTCTCGGA   60
KX257272_H31          AAGGTTAAAAGGAACAGTTGGGGGTATTCATATTCACTAGTTAGAGGTGAAATTCTCGGA   60
KX257273_H38          AAGGTTAAAAGGAACAGTTGGGGGTATTCATATTCACTAGTTAGAGGTGAAATTCTCGGA   60
KX257275_H46          AAGGTTAAAAGGAACAGTTGGGGGTATTCATATTCACTAGTTAGAGGTGAAATTCTCGGA   60
KX257278_H366         AAGGTTAAAAGGAACAGTTGGGGGTATTCATATTCACTAGTTAGAGGTGAAATTCTCGGA   60
KX257280_H496         AAGGTTAAAAGGAACAGTTGGGGGTATTCATATTCACTAGTTAGAGGTGAAATTCTCGGA   60
KX257281_H543         AAGGTTAAAAGGAACAGTTGGGGGTATTCATATTCACTAGTTAGAGGTGAAATTCTCGGA   60
KX257282_H621         AAGGTTAAAAGGAACAGTTGGGGGTATTCATATTCACTAGTTAGAGGTGAAATTCTCGGA   60
MK801358_Bsp_ST1      AAGGTTAAAAGGAACAGTTGGGGGTATTCATATTCACTAGTTAGAGGTGAAATTCTCGGA   60
KX257269_S03          AGATTAAAAGGAACAGTCGGGGCGTATTCATATTCACTAGTTAGAGGTGAAATTCTCGGA   60
MK801414_Bsp_ST5      AAGATTAAAAGGAACAGTTGGGGGTATTCATATTCAATAGTTAGAGGTGAAATTCTCGGA   60
KX257267_S01          AGATTAAAAGGAACAGTCGGGGCGTATTCATATTCAATAGTTAGAGGCGAAATTCTCGGA   60
KX257268_S02          AGATTAAAAGGAACAGTCGGGGCGTATTCATATTCAATAGTTAGAGGCGAAATTCTCGGA   60
                      *   * **  *        *** ************* ***  ***** ********  **

KX257266_B01          TTTATGGAAGATGAACTAGTGCGAAAGCATTTACCAAGGATGTTTTCATTAATCAAGAAC   120
MH507326_Bsp_ST10     TTTATGGAAGATGAACTAGTGCGAAAGCATTTACCAAGGATGTTTTCATTAATCAAGAAC   120
KX257270_S06          TCTATGGAAGATGAACTAGTGCGAAAGCATTTACCAAGGATGTTTTCATTAATCAAGAAC   120
KX257274_H40          TTTATGGAAGATGAACAAGTGCGAAAGCATTTACCAAGGATGTTTTCATTAATCAAGAAC   120
KX257283_H177         TTTATGGAAGATGAACAAGTGCGAAAGCATTTACCAAGGATGTTTTCATTAATCAAGAAC   120
KX257276_H212         TTTATGGAAGATGAACAAGTGCGAAAGCATTTACCAAGGATGTTTTCATTAATCAAGAAC   120
KX257277_H216         TTTATGGAAGATGAACAAGTGCGAAAGCATTTACCAAGGATGTTTTCATTAATCAAGAAC   120
KX257279_H495         TTTATGGAAGATGAACAAGTGCGAAAGCATTTACCAAGGATGTTTTCATTAATCAAGAAC   120
MK801403_Bsp_ST3      TTTATGGAAGATGAACAAGTGCGAAAGCATTTACCAAGGATGTTTTCATTAATCAAGAAC   120
AF408427_Bsp-ST7      TTTATGGAAGATGAACTAGTGCGAAAGCATTTACCAAGGATGTTTTCATTAATCAAGAAC   120
KX257271_H09          TTTATGGAAGATGAACAAGTGCGAAAGCATTTACCAAGGATGTTTTCATTAATCAAGAAC   120
KX257272_H31          TTTATGGAAGATGAACAAGTGCGAAAGCATTTACCAAGGATGTTTTCATTAATCAAGAAC   120
KX257273_H38          TTTATGGAAGATGAACAAGTGCGAAAGCATTTACCAAGGATGTTTTCATTAATCAAGAAC   120
KX257275_H46          TTTATGGAAGATGAACAAGTGCGAAAGCATTTACCAAGGATGTTTTCATTAATCAAGAAC   120
KX257278_H366         TTTATGGAAGATGAACAAGTGCGAAAGCATTTACCAAGGATGTTTTCATTAATCAAGAAC   120
KX257280_H496         TTTATGGAAGATGAACAAGTGCGAAAGCATTTACCAAGGATGTTTTCATTAATCAAGAAC   120
KX257281_H543         TTTATGGAAGATGAACAAGTGCGAAAGCATTTACCAAGGATGTTTTCATTAATCAAGAAC   120
KX257282_H621         TTTATGGAAGATGAACAAGTGCGAAAGCATTTACCAAGGATGTTTTCATTAATCAAGAAC   120
MK801358_Bsp_ST1      TTTATGGAAGATGAACAAGTGCGAAAGCATTTACCAAGGATGTTTTCATTAATCAAGAAC   120
KX257269_S03          TCTATGGAAGATGAACTAGTGCGAAAGCATTTACCAAGGATGTTTTCATTAATCAAGAAC   120
MK801414_Bsp_ST5      TTTATGGAAGATGAACTAGTGCGAAAGCATTTACCAAGGATGTTTTCATTAATCAAGAAC   120
KX257267_S01          TCTATGGAAGATGAACTAGTGCGAAAGCATTTACCAAGGATGTTTTCATTAATCAAGAAC   120
KX257268_S02          TCTATGGAAGATGAACTAGTGCGAAAGCATTTACCAAGGATGTTTTCATTAATCAAGAAC   120
                      * ************** *******************************************

KX257266_B01          GAAAGTTAGGGGATCGAAGAGGCTTAGATACCCTCGTAGTCTTAACTATAAACGATACCG   180
MH507326_Bsp_ST10     GAAAGTTAGGGGATCGAAGAGGCTTAGATACCCTCGTAGTCTTAACTATAAACGATACCG   180
KX257270_S06          GAAAGTTAGGGGATCGAAGAGGATTAGATACCCTCGTAGTCTTAACTATAAACGATACCG   180
KX257274_H40          GAAAGTTAGGGGATCGAAGAGGATTAGATACCCTCGTAGTCTTAACTATAAACGATACCG   180
KX257283_H177         GAAAGTTAGGGGATCGAAGAGGATTAGATACCCTCGTAGTCTTAACTATAAACGATACCG   180
KX257276_H212         GAAAGTTAGGGGATCGAAGAGGATTAGATACCCTCGTAGTCTTAACTATAAACGATACCG   180
KX257277_H216         GAAAGTTAGGGGATCGAAGAGGATTAGATACCCTCGTAGTCTTAACTATAAACGATACCG   180
KX257279_H495         GAAAGTTAGGGGATCGAAGAGGATTAGATACCCTCGTAGTCTTAACTATAAACGATACCG   180
MK801403_Bsp_ST3      GAAAGTTAGGGGATCGAAGAGGATTAGATACCCTCGTAGTCTTAACTATAAACGATACCG   180
AF408427_Bsp-ST7      GAAAGCTAGGGGATCAAAGAGGATTAGATACCCTCGTAGTCTTAGCTATAAACGATACCG   180
KX257271_H09          GAAAGCTAGGGGATCGAAGAGGATTAGATACCCTCGTAGTCTTAGCTATAAACGATACCG   180
KX257272_H31          GAAAGCTAGGGGATCGAAGAGGATTAGATACCCTCGTAGTCTTAGCTATAAACGATACCG   180
KX257273_H38          GAAAGCTAGGGGATCGAAGAGGATTAGATACCCTCGTAGTCTTAGCTATAAACGATACCG   180
KX257275_H46          GAAAGCTAGGGGATCGAAGAGGATTAGATACCCTCGTAGTCTTAGCTATAAACGATACCG   180
KX257278_H366         GAAAGCTAGGGGATCGAAGAGGATTAGATACCCTCGTAGTCTTAGCTATAAACGATACCG   180
KX257280_H496         GAAAGCTAGGGGATCGAAGAGGATTAGATACCCTCGTAGTCTTAGCTATAAACGATACCG   180
KX257281_H543         GAAAGCTAGGGGATCGAAGAGGATTAGATACCCTCGTAGTCTTAGCTATAAACGATACCG   180
KX257282_H621         GAAAGCTAGGGGATCGAAGAGGATTAGATACCCTCGTAGTCTTAGCTATAAACGATACCG   180
MK801358_Bsp_ST1      GAAAGCTAGGGGATCGAAGAGGATTAGATACCCTCGTAGTCTTAGCTATAAACGATACCG   180
KX257269_S03          GAAAGCTAGGGGATCGAAGAGGATTAGATACCCTCGTAGTCTTAGCTATAAACGATACCG   180
MK801414_Bsp_ST5      GAAAGCTAGGGGATCGAAGAGGCTTAGATACCCTCGTAGTCTTAGCTATAAACTATACCG   180
KX257267_S01          GAAAGCTAGGGGATCGAAGAGGCTTAGATACCCTCGTAGTCTTAGCTATAAACTATACCG   180
KX257268_S02          GAAAGCTAGGGGATCGAAGAGGCTTAGATACCCTCGTAGTCTTAGCTATAAACTATACCG   180
                      ***** ********* ****** ********************* ******** ******
```

```
KX257266_B01            ACTAGGGGTTAGTGGAGGTC-ATT-TGTCTCTACTAGTACCTTATGAGAAATCAAAGTCT   238
MH507326_Bsp_ST10       ACTAGGGGTTAGTGGAGGTC-ATT-TGTCTCTACTAGTACCTTATGAGAAATCAAAGTCT   238
KX257270_S06            ACTAGAGGTTAGTGAAGGTC-ATTGTGTCTTGACTAGTATCTTATGAGAAATCAAAGTCT   239
KX257274_H40            ACTAGAGGTTAGTGAAGGTC-ATTGTGTCTTGACTAGTATCTTATGAGAAATCAAAGTCT   239
KX257283_H177           ACTAGAGGTTAGTGAAGGTC-ATTGTGTCTTGACTAGTATCTTATGAGAAATCAAAGTCT   239
KX257276_H212           ACTAGAGGTTAGTGAAGGTC-ATTGTGTCTTGACTAGTATCTTATGAGAAATCAAAGTCT   239
KX257277_H216           ACTAGAGGTTAGTGAAGGTC-ATTGTGTCTTGACTAGTATCTTATGAGAAATCAAAGTCT   239
KX257279_H495           ACTAGAGGTTAGTGAAGGTC-ATTGTGTCTTGACTAGTATCTTATGAGAAATCAAAGTCT   239
MK801403_Bsp_ST3        ACTAGAGGTTAGTGAAGGTC-ATTGTGTCTTGACTAGTATCTTATGAGAAATCAAAGTCT   239
AF408427_Bsp-ST7        ACTAGGAGTTAGTAGATGATCCAATGGTGTCTATTAGTACCTTATGAGAAATCAAAGTCT   240
KX257271_H09            ACTAGGGGTTAGTAGAGGTC-AAAGTGTCTTTATTAGTACCTTATGAGAAATCAAAGTCT   239
KX257272_H31            ACTAGGGGTTAGTAGAGGTC-AAAGTGTCTTTATTAGTACCTTATGAGAAATCAAAGTCT   239
KX257273_H38            ACTAGGGGTTAGTAGAGGTC-AAAGTGTCTTTATTAGTACCTTATGAGAAATCAAAGTCT   239
KX257275_H46            ACTAGGGGTTAGTAGAGGTC-AAAGTGTCTTTATTAGTACCTTATGAGAAATCAAAGTCT   239
KX257278_H366           ACTAGGGGTTAGTAGAGGTC-AAAGTGTCTTTATTAGTACCTTATGAGAAATCAAAGTCT   239
KX257280_H496           ACTAGGGGTTAGTAGAGGTC-AAAGTGTCTTTATTAGTACCTTATGAGAAATCAAAGTCT   239
KX257281_H543           ACTAGGGGTTAGTAGAGGTC-AAAGTGTCTTTATTAGTACCTTATGAGAAATCAAAGTCT   239
KX257282_H621           ACTAGGGGTTAGTAGAGGTC-AAAGTGTCTTTATTAGTACCTTATGAGAAATCAAAGTCT   239
MK801358_Bsp_ST1        ACTAGGGGTTAGTAGAGGTC-AAAGTGTCTTTATTAGTACCTTATGAGAAATCAAAGTCT   239
KX257269_S03            ACTAGGGGTTAGTAGAGGTC-AAAGTGTCTTTATTAGTACCTTATGAGAAATCAAAGTCT   239
MK801414_Bsp_ST5        ACTAGGGGTTAGTAGAAGTC-ATTGTGTTTCTATTAGTACCTTATGAGAAATCAAAGTCT   239
KX257267_S01            ACTAGGGGTTAGTAGAAGTC-ATTGTGTTTCTATTAGTACCTTATGAGAAATCAAAGTCT   239
KX257268_S02            ACTAGGGGTTAGTAGAAGTC-ATTGTGTTTCTATTAGTACCTTATGAGAAATCAAAGTCT   239
                        *****  ******  * *        ** *  * ***** ********************

KX257266_B01            TTGGGTTCCGGGGGGAGTATGGTCGCAAGGCTGAAACTTAAAGGAATTGACGGAAGGGCA   298
MH507326_Bsp_ST10       TTGGGTTCCGGGGGGAGTATGGTCGCAAGGCTGAAACTTAAAGGAATTGACGGAAGGGCA   298
KX257270_S06            TTGGGTTCCGGGGGGAGTATGGTCGCAAGGCTGAAACTTAAAGGAATTGACGGAAGGGCA   299
KX257274_H40            TTGGGTTCCGGGGGGAGTATGGTCGCAAGGCTGAAACTTAAAGGAATTGACGGAAGGGCA   299
KX257283_H177           TTGGGTTCCGGGGGGAGTATGGTCGCAAGGCTGAAACTTAAAGGAATTGACGGAAGGGCA   299
KX257276_H212           TTGGGTTCCGGGGGGAGTATGGTCGCAAGGCTGAAACTTAAAGGAATTGACGGAAGGGCA   299
KX257277_H216           TTGGGTTCCGGGGGGAGTATGGTCGCAAGGCTGAAACTTAAAGGAATTGACGGAAGGGCA   299
KX257279_H495           TTGGGTTCCGGGGGGAGTATGGTCGCAAGGCTGAAACTTAAAGGAATTGACGGAAGGGCA   299
MK801403_Bsp_ST3        TTGGGTTCCGGGGGGAGTATGGTCGCAAGGCTGAAACTTAAAGGAATTGACGGAAGGGCA   299
AF408427_Bsp-ST7        TTGGGTTCCGGGGGGAGTATGGTCGCAAGGCTGAAACTTAAAGGAATTGACGGAAGGGCA   300
KX257271_H09            TTGGGTTCCGGGGGGAGTATGGTCGCAAGGCTGAAACTTAAAGGAATTGACGGAAGGGCA   299
KX257272_H31            TTGGGTTCCGGGGGGAGTATGGTCGCAAGGCTGAAACTTAAAGGAATTGACGGAAGGGCA   299
KX257273_H38            TTGGGTTCCGGGGGGAGTATGGTCGCAAGGCTGAAACTTAAAGGAATTGACGGAAGGGCA   299
KX257275_H46            TTGGGTTCCGGGGGGAGTATGGTCGCAAGGCTGAAACTTAAAGGAATTGACGGAAGGGCA   299
KX257278_H366           TTGGGTTCCGGGGGGAGTATGGTCGCAAGGCTGAAACTTAAAGGAATTGACGGAAGGGCA   299
KX257280_H496           TTGGGTTCCGGGGGGAGTATGGTCGCAAGGCTGAAACTTAAAGGAATTGACGGAAGGGCA   299
KX257281_H543           TTGGGTTCCGGGGGGAGTATGGTCGCAAGGCTGAAACTTAAAGGAATTGACGGAAGGGCA   299
KX257282_H621           TTGGGTTCCGGGGGGAGTATGGTCGCAAGGCTGAAACTTAAAGGAATTGACGGAAGGGCA   299
MK801358_Bsp_ST1        TTGGGTTCCGGGGGGAGTATGGTCGCAAGGCTGAAACTTAAAGGAATTGACGGAAGGGCA   299
KX257269_S03            TTGGGTTCCGGGGGGAGTATGGTCGCAAGGCTGAAACTTAAAGGAATTGACGGAAGGGCA   299
MK801414_Bsp_ST5        TTGGGTTCCGGGGGGAGTATGGTCGCAAGGCTGAAACTTAAAGGAATTGACGGAAGGGCA   299
KX257267_S01            TTGGGTTCCGGGGGGAGTATGGTCGCAAGGCTGAAACTTAAAGGAATTGACGGAAGGGCA   299
KX257268_S02            TTGGGTTCCGGGGGGAGTATGGTCGCAAGGCTGAAACTTAAAGGAATTGACGGAAGGGCA   299
                        ************************************************************

KX257266_B01            CCACCAGGAGTGGAGCCTGCGGCTTAATTTGACTCAACACGGGGAAACTTACCAGGTCCA   358
MH507326_Bsp_ST10       CCACCAGGAGTGGAGCCTGCGGCTTAATTTGACTCAACACGGGGAAACTTACCAGGTCCA   358
KX257270_S06            CCACCAGGAGTGGAGCCTGCGGCTTAATTTGACTCAACACGGGAAAACTTACCAGGTCCA   359
KX257274_H40            CCACCAGGAGTGGAGCCTGCGGCTTAATTTGACTCAACACGGGAAAACTTACCAGGTCCA   359
KX257283_H177           CCACCAGGAGTGGAGCCTGCGGCTTAATTTGACTCAACACGGGAAAACTTACCAGGTCCA   359
KX257276_H212           CCACCAGGAGTGGAGCCTGCGGCTTAATTTGACTCAACACGGGAAAACTTACCAGGTCCA   359
KX257277_H216           CCACCAGGAGTGGAGCCTGCGGCTTAATTTGACTCAACACGGGAAAACTTACCAGGTCCA   359
KX257279_H495           CCACCAGGAGTGGAGCCTGCGGCTTAATTTGACTCAACACGGGAAAACTTACCAGGTCCA   359
MK801403_Bsp_ST3        CCACCAGGAGTGGAGCCTGCGGCTTAATTTGACTCAACACGGGAAAACTTACCAGGTCCA   359
AF408427_Bsp-ST7        CCACCAGGAGTGGAGCCTGCGGCTTAATTTGACTCAACACGGGGAAACTTACCAGGTCCA   360
KX257271_H09            CCACCAGGAGTGGAGCCTGCGGCTTAATTTGACTCAACACGGGGAAACTTACCAGGTCCA   359
KX257272_H31            CCACCAGGAGTGGAGCCTGCGGCTTAATTTGACTCAACACGGGGAAACTTACCAGGTCCA   359
KX257273_H38            CCACCAGGAGTGGAGCCTGCGGCTTAATTTGACTCAACACGGGGAAACTTACCAGGTCCA   359
KX257275_H46            CCACCAGGAGTGGAGCCTGCGGCTTAATTTGACTCAACACGGGGAAACTTACCAGGTCCA   359
KX257278_H366           CCACCAGGAGTGGAGCCTGCGGCTTAATTTGACTCAACACGGGGAAACTTACCAGGTCCA   359
KX257280_H496           CCACCAGGAGTGGAGCCTGCGGCTTAATTTGACTCAACACGGGGAAACTTACCAGGTCCA   359
KX257281_H543           CCACCAGGAGTGGAGCCTGCGGCTTAATTTGACTCAACACGGGGAAACTTACCAGGTCCA   359
KX257282_H621           CCACCAGGAGTGGAGCCTGCGGCTTAATTTGACTCAACACGGGGAAACTTACCAGGTCCA   359
MK801358_Bsp_ST1        CCACCAGGAGTGGAGCCTGCGGCTTAATTTGACTCAACACGGGGAAACTTACCAGGTCCA   359
KX257269_S03            CCACCAGGAGTGGAGCCTGCGGCTTAATTTGACTCAACACGGGGAAACTTACCAGGTCCA   359
MK801414_Bsp_ST5        CCACCAGGAGTGGAGCCTGCGGCTTAATTTGACTCAACACGGGGAAACTTACCAGGTCCA   359
KX257267_S01            CCACCAGGAGTGGAGCCTGCGGCTTAATTTGACTCAACACGGGGAAACTTACCAGGTCCA   359
KX257268_S02            CCACCAGGAGTGGAGCCTGCGGCTTAATTTGACTCAACACGGGGAAACTTACCAGGTCCA   359
                        ******************************************* ****************
```

```
KX257266_B01           GACATAGGAAGGATAGACAGAAC-AAAGCTCTTTCTTGATTCTATGGGTGGTGGTGCATG   417
MH507326_Bsp_ST10      GACATAGGAAGGATAGACAGAAC-AAAGCTCTTTCTTGATTCTATGGGTGGTGGTGCATG   417
KX257270_S06           GACATAGGAAGGATAGACAGATT-AAAGCTCTTTCTTGATTCTATGGGTGGTGGTGCATG   418
KX257274_H40           GACATAGGAAGGATAGACAGATT-AAAGCTCTTTCTTGATTCTATGGGTGGTGGTGCATG   418
KX257283_H177          GACATAGGAAGGATAGACAGATT-AAAGCTCTTTCTTGATTCTATGGGTGGTGGTGCATG   418
KX257276_H212          GACATAGGAAGGATAGACAGATT-AAAGCTCTTTCTTGATTCTATGGGTGGTGGTGCATG   418
KX257277_H216          GACATAGGAAGGATAGACAGATT-AAAGCTCTTTCTTGATTCTATGGGTGGTGGTGCATG   418
KX257279_H495          GACATAGGAAGGATAGACAGATT-AAAGCTCTTTCTTGATTCTATGGGTGGTGGTGCATG   418
MK801403_Bsp_ST3       GACATAGGAAGGATAGACAGATT-AAAGCTCTTTCTTGATTCTATGGGTGGTGGTGCATG   418
AF408427_Bsp-ST7       GACATAGGAAGGATTGACAGATTGATAGCTCTTTCTTGATTCTATGGGTGGTGGTGCATG   420
KX257271_H09           GACATAGGAAGGATTGACAGATTGATAGCTCTTTCTTGATTCTATGGGTGGTGGTGCATG   419
KX257272_H31           GACATAGGAAGGATTGACAGATTGATAGCTCTTTCTTGATTCTATGGGTGGTGGTGCATG   419
KX257273_H38           GACATAGGAAGGATTGACAGATTGATAGCTCTTTCTTGATTCTATGGGTGGTGGTGCATG   419
KX257275_H46           GACATAGGAAGGATTGACAGATTGATAGCTCTTTCTTGATTCTATGGGTGGTGGTGCATG   419
KX257278_H366          GACATAGGAAGGATTGACAGATTGATAGCTCTTTCTTGATTCTATGGGTGGTGGTGCATG   419
KX257280_H496          GACATAGGAAGGATTGACAGATTGATAGCTCTTTCTTGATTCTATGGGTGGTGGTGCATG   419
KX257281_H543          GACATAGGAAGGATTGACAGATTGATAGCTCTTTCTTGATTCTATGGGTGGTGGTGCATG   419
KX257282_H621          GACATAGGAAGGATTGACAGATTGATAGCTCTTTCTTGATTCTATGGGTGGTGGTGCATG   419
MK801358_Bsp_ST1       GACATAGGAAGGATTGACAGATTGATAGCTCTTTCTTGATTCTATGGGTGGTGGTGCATG   419
KX257269_S03           GACATAGGAAGGATTGACAGATTGATAGCTCTTTCTTGATTCTATGGGTGGTGGTGCATG   419
MK801414_Bsp_ST5       GACATAGGAAGGATTGACAGATTGATAGCTCTTTCTTGATTCTATGGGTGGTGGTGCATG   419
KX257267_S01           GACATAGGAAGGATTGACAGATTGATAGCTCTTTCTTGATTCTATGGGTGGTGGTGCATG   419
KX257268_S02           GACATAGGAAGGATTGACAGATTGATAGCTCTTTCTTGATTCTATGGGTGGTGGTGCATG   419
                       ************** ******   * **********************************

KX257266_B01           GCCGTTCTTAGTTGGTGGATTGATTTGTCAGGCTAATTCCGATAACGAACGAGACCTCCG   477
MH507326_Bsp_ST10      GCCGTTCTTAGTTGGTGGATTGATTTGTCAGGCTAATTCCGATAACGAACGAGACCTCCG   477
KX257270_S06           GCCGTTCTTAGTTGGTGGATTGATTTGTCAGGCTAATTCCGATAACGAACGAGACCTCCG   478
KX257274_H40           GCCGTTCTTAGTTGGTGGATTGATTTGTCAGGCTAATTCCGATAACGAACGAGACCTCCG   478
KX257283_H177          GCCGTTCTTAGTTGGTGGATTGATTTGTCAGGCTAATTCCGATAACGAACGAGACCTCCG   478
KX257276_H212          GCCGTTCTTAGTTGGTGGATTGATTTGTCAGGCTAATTCCGATAACGAACGAGACCTCCG   478
KX257277_H216          GCCGTTCTTAGTTGGTGGATTGATTTGTCAGGCTAATTCCGATAACGAACGAGACCTCCG   478
KX257279_H495          GCCGTTCTTAGTTGGTGGATTGATTTGTCAGGCTAATTCCGATAACGAACGAGACCTCCG   478
MK801403_Bsp_ST3       GCCGTTCTTAGTTGGTGGATTGATTTGTCAGGCTAATTCCGATAACGAACGAGACCTCCG   478
AF408427_Bsp-ST7       GCCGTTCTTAGTTGGTGGAGTGATTTGTCTGGTTTATTCCGATAACGAACGAGACTTCCG   480
KX257271_H09           GCCGTTCTTAGTTGGTGGAGTGATTTGTCTGGCTAATTCCGATAACGAACGAGACCTCCG   479
KX257272_H31           GCCGTTCTTAGTTGGTGGAGTGATTTGTCTGGCTAATTCCGATAACGAACGAGACCTCCG   479
KX257273_H38           GCCGTTCTTAGTTGGTGGAGTGATTTGTCTGGCTAATTCCGATAACGAACGAGACCTCCG   479
KX257275_H46           GCCGTTCTTAGTTGGTGGAGTGATTTGTCTGGCTAATTCCGATAACGAACGAGACCTCCG   479
KX257278_H366          GCCGTTCTTAGTTGGTGGAGTGATTTGTCTGGCTAATTCCGATAACGAACGAGACCTCCG   479
KX257280_H496          GCCGTTCTTAGTTGGTGGAGTGATTTGTCTGGCTAATTCCGATAACGAACGAGACCTCCG   479
KX257281_H543          GCCGTTCTTAGTTGGTGGAGTGATTTGTCTGGCTAATTCCGATAACGAACGAGACCTCCG   479
KX257282_H621          GCCGTTCTTAGTTGGTGGAGTGATTTGTCTGGCTAATTCCGATAACGAACGAGACCTCCG   479
MK801358_Bsp_ST1       GCCGTTCTTAGTTGGTGGAGTGATTTGTCTGGCTAATTCCGATAACGAACGAGACCTCCG   479
KX257269_S03           GCCGTTCTTAGTTGGTGGAGTGATTTGTCTGGCTAATTCCGATAACGAACGAGACCTCCG   479
MK801414_Bsp_ST5       GCCGTTCTTAGTTGGTGGAGTGATTTGTCTGGTTAATTCCGATAACGAACGAGACCTCCG   479
KX257267_S01           GCCGTTCTTAGTTGGTGGAGTGATTTGTCTGGTTAATTCCGATAACGAACGAGACCTCCG   479
KX257268_S02           GCCGTTCTTAGTTGGTGGAGTGATTTGTCTGGTTAATTCCGATAACGAACGAGACCTCCG   479
                       ******************* ********* ** * ******************** ****

KX257266_B01           CCTGCTAAATAGAAAATTATAATTCGTTATAGT-----------TTTCTTCTTAGAGGGA   526
MH507326_Bsp_ST10      CCTGCTAAATAGAAAATTATAATTCGTTATAGT-----------TTTCTTCTTAGAGGGA   526
KX257270_S06           CCTGCTAAATGGTTTTCTATAGTATTCTATAGG-----------AATCCTCTTAGAGGGA   527
KX257274_H40           CCTGCTAAATGGTTTTCTATAGTATTCTATAGG-----------AATCCTCTTAGAGGGA   527
KX257283_H177          CCTGCTAAATGGTTTTCTATAGTATTCTATAGG-----------AATCCTCTTAGAGGGA   527
KX257276_H212          CCTGCTAAATGGTTTTCTATAGTATTCTATAGG-----------AATCCTCTTAGAGGGA   527
KX257277_H216          CCTGCTAAATGGTTTTCTATAGTATTCTATAGG-----------AATCCTCTTAGAGGGA   527
KX257279_H495          CCTGCTAAATGGTTTTCTATAGTATTCTATAGG-----------AATCCTCTTAGAGGGA   527
MK801403_Bsp_ST3       CCTGCTAAATGGTTTTCTATAGTATTCTATAGG-----------AATCCTCTTAGAGGGA   527
AF408427_Bsp-ST7       CCTATTAGTTGGATGAAATGGGATTTTTAGCCCCATTATTTTTTCATCAGCTTAGAGGGA   540
KX257271_H09           CCTTTAACTAGTGACGTGTATT------------GTGATATGCGTTGCTTCTTATAGGGA   527
KX257272_H31           CCTTTAACTAGTGACGTGTATT------------GTGATATGCGTTGCTTCTTATAGGGA   527
KX257273_H38           CCTTTAACTAGTGACGTGTATT------------GTGATATGCGTTGCTTCTTATAGGGA   527
KX257275_H46           CCTTTAACTAGTGACGTGTATT------------GTGATATGCGTTGCTTCTTATAGGGA   527
KX257278_H366          CCTTTAACTAGTGACGTGTATT------------GTGATATGCGTTGCTTCTTATAGGGA   527
KX257280_H496          CCTTTAACTAGTGACGTGTATT------------GTGATATGCGTTGCTTCTTATAGGGA   527
KX257281_H543          CCTTTAACTAGTGACGTGTATT------------GTGATATGCGTTGCTTCTTATAGGGA   527
KX257282_H621          CCTTTAACTAGTGACGTGTATT------------GTGATATGCGTTGCTTCTTATAGGGA   527
MK801358_Bsp_ST1       CCTTTAACTAGTGACGTGTATT------------GTGATATGCGTTGCTTCTTATAGGGA   527
KX257269_S03           CCTTTAACTAGTGACGTGTATT------------GTGATATGCGTTGCTTCTTATAGGGA   527
MK801414_Bsp_ST5       CCTGCTAAATAGTATATATTATTT----------TTAATAATGTATACTTCTTAGAGGGA   529
KX257267_S01           CCTGCTAAATAGTATATATAATTTT---------GTAATAATGTATACTTCTTAGAGGGA   530
KX257268_S02           CCTGCTAAATAGTATATATTATTTT---------GTAATAATGTATACTTCTTAGAGGGA   530
                       ***   *                                        *  **** *****
```

```
KX257266_B01          CACTA-TACATCAAGTGTAGGGAAGCTGGAGGCAATAACAGGTCTGTGATGCCCTTAGAT   585
MH507326_Bsp_ST10     CACTA-TACATCAAGTGTAGGGAAGCTGGAGGCAATAACAGGTCTGTGATGCCCTTAGAT   585
KX257270_S06          CACTA-TATATAAAGTATAGGGAAGCTGGAGGCAATAACAGGTCTGTGATGCCCTTAGAT   586
KX257274_H40          CACTA-TACATAAAGTGTAGGGAAGCTGGAGGCAATAACAGGTCTGTGATGCCCTTAGAT   586
KX257283_H177         CACTA-TACATAAAGTGTAGGGAAGCTGGAGGCAATAACAGGTCTGTGATGCCCTTAGAT   586
KX257276_H212         CACTA-TACATAAAGTGTAGGGAAGCTGGAGGCAATAACAGGTCTGTGATGCCCTTAGAT   586
KX257277_H216         CACTA-TACATAAAGTGTAGGGAAGCTGGAGGCAATAACAGGTCTGTGATGCCCTTAGAT   586
KX257279_H495         CACTA-TACATAAAGTGTAGGGAAGCTGGAGGCAATAACAGGTCTGTGATGCCCTTAGAT   586
MK801403_Bsp_ST3      CACTA-TACATAAAGTATAGGGAAGCTGGAGGCAATAACAGGTCTGTGATGCCCTTAGAT   586
AF408427_Bsp-ST7      CACTGTGCGTTTGAGTACAGGGAAGCTGGAGGCAATAACAGGTCTGTGATGCCCTTAGAT   600
KX257271_H09          CACTATATG-TAAAATGTAGGGAAGCTGGAGGCAATAACAGGTCTGTGATGCCCTTAGAT   586
KX257272_H31          CACTATATG-TAAAATGTAGGGAAGCTGGAGGCAATAACAGGTCTGTGATGCCCTTAGAT   586
KX257273_H38          CACTATATG-TAAAATGTAGGGAAGCTGGAGGCAATAACAGGTCTGTGATGCCCTTAGAT   586
KX257275_H46          CACTATATG-TAAAATGTAGGGAAGCTGGAGGCAATAACAGGTCTGTGATGCCCTTAGAT   586
KX257278_H366         CACTATATG-TAAAATGTAGGGAAGCTGGAGGCAATAACAGGTCTGTGATGCCCTTAGAT   586
KX257280_H496         CACTATATG-TAAAATGTAGGGAAGCTGGAGGCAATAACAGGTCTGTGATGCCCTTAGAT   586
KX257281_H543         CACTATATG-TAAAATGTAGGGAAGCTGGAGGCAATAACAGGTCTGTGATGCCCTTAGAT   586
KX257282_H621         CACTATATG-TAAAATGTAGGGAAGCTGGAGGCAATAACAGGTCTGTGATGCCCTTAGAT   586
MK801358_Bsp_ST1      CACTATATG-TAAAATGTAGGGAAGCTGGAGGCAATAACAGGTCTGTGATGCCCTTAGAT   586
KX257269_S03          CACTATATG-TAAAATGTAGGGAAGCTGGAGGCAATAACAGGTCTGTGATGCCCTTAGAT   586
MK801414_Bsp_ST5      CACTATACGTATGAGTGTAGGGAAGCTGGAGGCAATAACAGGTCTGTGATGCCCTTAGAT   589
KX257267_S01          CACTATATGTATGAGTGTAGGGAAGCTGGAGGCAATAACAGGTCTGTGATGCCCTTAGAT   590
KX257268_S02          CACTATATGTATGAGTGTAGGGAAGCTGGAGGCAATAACAGGTCTGTGATGCCCTTAGAT   590
                      ****         * *  ************ *****************************

KX257266_B01          GTCCTGGGCTGCACGCGCGCGA   607
MH507326_Bsp_ST10     GTCCTGGGCTGCACGCGCGCGA   607
KX257270_S06          GTCCTGGGCTGCACGCGCGCGA   608
KX257274_H40          GTCCTGGGCTGCACGCGCGCGA   608
KX257283_H177         GTCCTGGGCTGCACGCGCGCGA   608
KX257276_H212         GTCCTGGGCTGCACGCGCGCGA   608
KX257277_H216         GTCCTGGGCTGCACGCGCGCGA   608
KX257279_H495         GTCCTGGGCTGCACGCGCGCGA   608
MK801403_Bsp_ST3      GTCCTGGGCTGCACGCGCGCGA   608
AF408427_Bsp-ST7      GTTCTGGGCTGAACGCGCGCGA   622
KX257271_H09          GTTCTGGGCTGCACGCGCGCGA   608
KX257272_H31          GTTCTGGGCTGCACGCGCGCGA   608
KX257273_H38          GTTCTGGGCTGCACGCGCGCGA   608
KX257275_H46          GTTCTGGGCTGCACGCGCGCGA   608
KX257278_H366         GTTCTGGGCTGCACGCGCGCGA   608
KX257280_H496         GTTCTGGGCTGCACGCGCGCGA   608
KX257281_H543         GTTCTGGGCTGCACGCGCGCGA   608
KX257282_H621         GTTCTGGGCTGCACGCGCGCGA   608
MK801358_Bsp_ST1      GTTCTGGGCTGCACGCGCGCGA   608
KX257269_S03          GTTCTGGGCTGCACGCGCGCGA   608
MK801414_Bsp_ST5      GTTCTGGGCTGCACGCGCGCGA   611
KX257267_S01          GTTCTGGGCTGCACGCGCGCGA   612
KX257268_S02          GTTCTGGGCTGCACGCGCGCGA   612
                      ** ******** **********
```

Figure S1: Multiple sequence alignment of 18S rDNA sequences of *Blastocystis* spp. subtypes. Sequences described in this study are: B01 (acession number: KX257266), H09 (KX257271), H31 (KX257272), H38 (KX257273), H40 (KX257274), H46 (KX257275), H177 (KX257283), H212 (KX257276), H216 (KX257277), H366 (KX257278), H495 (KX257279), H496 (KX257280), H543 (KX257281), H621 (KX257282), S01 (KX257267), S02 (KX257268), S03 (KX257269), and S06 (KX257270). Reference sequences of *Blastocystis* spp. subtypes are: Bsp_ST1 (MK801358), Bsp_ST3 (MK801403), Bsp_ST5 (MK801414), Bsp-ST7 (AF408427), and Bsp_ST10 (MH507326). Sequences were trimmed in order to allow proper comparison. Indel (insertion/deletion) events are indicated by traces (-). Conserved nucleotides in all samples are indicated by asteriscs (*).
